# Associations of substance use and positive coping behaviors with sleep quality during the COVID-19 pandemic

**DOI:** 10.3389/frsle.2024.1504489

**Published:** 2025-02-05

**Authors:** Jessica Laudie, Bethany L. Stangl, Tommy Gunawan, Melanie L. Schwandt, Cecilia Cheng, Amanda K. Gilmore, David Goldman, Vijay A. Ramchandani, Nancy Diazgranados, Jeremy W. Luk

**Affiliations:** ^1^National Institute on Alcohol Abuse and Alcoholism, Bethesda, MD, United States; ^2^Human Psychopharmacology Laboratory, National Institute on Alcohol Abuse and Alcoholism, Bethesda, MD, United States; ^3^Department of Psychology, The University of Hong Kong, Pokfulam, Hong Kong SAR, China; ^4^Department of Health Policy & Behavioral Sciences, School of Public Health, Georgia State University, Atlanta, GA, United States; ^5^National Center for Sexual Violence Prevention, Mark Chaffin Centers for Healthy Development, Georgia State University, Atlanta, GA, United States; ^6^Laboratory of Neurogenetics, National Institute on Alcohol Abuse and Alcoholism, Rockville, MD, United States

**Keywords:** alcohol, mental health, sleep quality, sleep medication, substance use, wellbeing

## Abstract

**Background:**

The COVID-19 pandemic may have negatively impacted individuals' sleep quality. In this study, we examined changes in sleep quality from before to during the COVID-19 pandemic by history of alcohol use disorder (AUD) and investigated the cross-sectional associations of substance use and positive coping behaviors with sleep quality domains.

**Methods:**

Data were drawn from the NIAAA COVID-19 Pandemic Impact on Alcohol Study which enrolled participants from June 2020 to December 2022. Participants (*N* = 373, 50.9% male, mean age = 43.7, 37.3% with AUD history) reported their sleep quality using the Pittsburgh Sleep Quality Index (PSQI) for the month prior to the pandemic and the past month before the time of assessment. Multilevel modeling and linear regression analyses were conducted.

**Results:**

Individuals with AUD history reported worse overall sleep quality than those without AUD history both before and during the pandemic. Individuals without AUD history reported an overall increase in PSQI scores (worsened sleep quality) across time. AUD history and substance use behaviors due to the pandemic were associated with worse sleep quality. Conversely, positive coping behaviors (e.g., take care of body, make time to relax, connect with others, multiple healthy behaviors) were associated with better sleep quality domains.

**Conclusions:**

AUD history, substance use, and positive coping behaviors were correlated with sleep quality during the pandemic. These findings may offer insight into behavioral targets to improve sleep quality in the context of significant stress exposure and can help improve preparedness for future public health crises.

## 1 Introduction

Severe acute respiratory syndrome coronavirus 2 (SARS-CoV-2) was a novel, highly contagious coronavirus that emerged in Wuhan, China in late 2019. SARS-CoV-2 is responsible for a series of acute respiratory infections known as coronavirus disease 2019, or COVID-19. COVID-19 rapidly spread across the globe, leading the World Health Organization to declare a pandemic on March 11, 2020. The biological impact of the virus was exacerbated by the psychosocial effects of the pandemic. Stringent efforts to mitigate the spread of the virus such as masking mandates, lockdowns, quarantine, and social distancing measures disrupted daily routines (Rajkumar, 2021; Richter et al., 2023; Guo et al., 2021). The COVID-19 pandemic was a significant stressor that affected many aspects of individuals' lives such as increased mental health symptoms and worsened quality of life (Luk et al., 2023b). With a rise in stress, worry, and fear during the COVID-19 pandemic, sleep problems pose a significant risk for worsening physical and psychological wellbeing. Notably, sleep is important for aspects of physical health that are affected by the COVID-19 virus, including immune system functioning, inflammation, and cardiovascular health (Besedovsky et al., 2019; Wolk et al., 2005).

A 2021 meta-analysis of studies from 13 countries found that 35.7% (95% Confidence Intervals: 29.4%−42.4%) all populations (including the general population, patients infected with COVID-19, and health care workers) reported sleep problems during the pandemic (Jahrami et al., 2021). At the population level, individuals generally reported more sleep problems in 2020 compared to 2018 (Hisler and Twenge, 2021). Sleep problems during the pandemic included shorter duration, inability to fall asleep, difficulty staying asleep, insomnia, and more days not rested (Hisler and Twenge, 2021; Marelli et al., 2021). While changes in sleep quality before and during the pandemic have been studied in various samples, few studies have examined the potential impact of alcohol use disorder (AUD) history. Current research has demonstrated that sleep disturbances are prevalent in AUD, both contributing to and being affected by AUD pathology (Koob and Colrain, 2020). Additionally, sleep disturbances arising from AUD can adversely affect individuals' wellbeing and health (Chakravorty et al., 2016).

Alcohol consumption itself has acute effects on sleep architecture. Multiple studies have shown that alcohol consumption decreases Rapid eye movement (REM) sleep and sleep onset latency while increases slow wave sleep, late-night disturbance, and wakefulness (Williams et al., 1983; Miyata et al., 2004; Arnedt et al., 2011). Chronic alcohol consumption as often seen in AUD may also alter sleep architecture (Koob and Colrain, 2020). A systematic review showed that mental health difficulties were associated with pandemic-related substance use (Roberts et al., 2021). However, limited research has specifically examined the associations between different types of substance use and sleep quality during the pandemic.

Positive coping behaviors, defined as behaviors that are used to cope with life stressors (e.g., taking care of their body, engaging in healthy behaviors, making time to relax, and connecting with others), may buffer against the impact of AUD on clinical outcomes such as depressive symptoms and problematic drinking (McCabe et al., 2024). In one study, Cheng and colleagues found that information coping style and information-seeking behavior, specifically seeking COVID-19 information via online media, were associated with greater susceptibility to emotional and sleep problems (Cheng et al., 2021). In another study, Johnson and colleagues found that control-oriented coping strategies (e.g., positive reappraisal, acceptance) were associated with a smaller increase in sleep disturbances (Johnson et al., 2022). These studies suggest that responses to pandemic stressors may have a significant impact on sleep quality and are moderated by how one copes with these stressors. According to a systematic review, positive coping behaviors among health care workers during the pandemic was associated with positive mental and psychological health outcomes (Labrague, 2021). Moreover, positive coping behaviors were associated with lower levels of psychological distress, anxiety, and depression (Labrague, 2021). Based on existing literature, positive coping behaviors may be associated with aspects of sleep quality during the pandemic.

Our prior research has examined changes in alcohol-related behaviors, quality of life, and loneliness from before to during the COVID-19 pandemic (Luk et al., 2023b, 2024). Extending these studies, the first goal of this study was to examine the impact of AUD history on changes in overall sleep quality from before to during the pandemic. The second goal of this study was to explore correlates of multiple domains of sleep quality. We treated substance use as negative coping strategies and examined their relationships with sleep quality. Moreover, we examined the associations between positive coping behaviors and sleep quality. We hypothesized that overall sleep quality would be worsened from before to during the pandemic, especially among those with AUD history. We also hypothesized that substance use behaviors would be associated with worse sleep quality, whereas positive coping behaviors would be associated with better sleep quality.

## 2 Methods

### 2.1 Participants and procedures

The study sample included participants (*N* = 373) who completed the baseline survey of the National Institute on Alcohol Abuse and Alcoholism (NIAAA) COVID-19 Pandemic Impact on Alcohol Study (C19-PIA Study). Participants were previously enrolled in the NIAAA Natural History Protocol between 2015 and 2022, a screening protocol that serves as a platform for participant recruitment into other NIAAA studies. The advantage of using this platform is that it provides rich screening data on participants. The Natural History Protocol assessed a wide range of behavioral and clinical characteristics among individuals across the spectrum of alcohol use. The time between completing the Natural History Protocol and enrollment into the C19-PIA Study varied across participants, with 42.6% (*n* = 159) enrolled in the C19-PIA Study <1 year after the Natural History Protocol, 17.4% (*n* = 65) within 1 year, 23.6% (*n* = 88) within 2–3 years, and 16.4% (*n* = 61) within 4 or more years. The World Health Organization declared a pandemic on March 11, 2020, and the baseline survey of the C19-PIA Study was administered between June 3, 2020, and December 30, 2022. To adjust for the potential confounding effect of enrollment timing, we created a categorical variable to capture five different enrollment phases that mapped onto different stages of the pandemic in terms of social distancing policies, surges of cases, availability of vaccines and other related factors: 19.3% (*n* = 72) enrolled between June 3, 2020 and July 31, 2020 (Phase I); 27.6% (*n* = 103) between August 1, 2020 and November 22, 2020 (Phase II); 24.4% (*n* = 91) between November 23, 2020 and February 28, 2021 (Phase III); 13.7% (*n* = 51) between March 1, 2021 and November 30, 2021 (Phase IV); 15.0% (*n* = 56) between December 1, 2021 and December 30 2022 (Phase V). AUD history was present among 37.3% (*n* = 139) of study participants. All participants provided verbal consent before they were enrolled in the C19 PIA Study. The C19-PIA study was approved by the NIH Intramural Institutional Review Board and is registered in clinicaltrials.gov (NCT04391816).

### 2.2 Measures

#### 2.2.1 Sleep quality

Sleep quality before and during the pandemic was assessed at the C19-PIA Study baseline survey using the Pittsburgh Sleep Quality Index (PSQI; Buysse et al., 1989). Participants were asked to complete the PSQI twice to report their sleep quality during two time periods: (1) the month prior to the pandemic, and (2) during the past month. The PSQI includes seven subscales of sleep duration, sleep disturbances, subjective sleep quality, use of medication for sleep, habitual sleep latency, sleep efficiency, and sleep dysfunction. For each subscale, a minimum score of 0 indicates better functioning while a maximum score of 3 indicates worse functioning. Total PSQI scores were calculated by summing all the subscales, with a higher score indicating worse sleep quality, and a score of 5 or higher indicating clinically significant sleep disturbance.

#### 2.2.2 AUD history and current alcohol consumption

AUD was diagnosed with the Structured Clinical Interview for Diagnostic and Statistical Manual of Mental Disorders (First, 2015) as part of the NIAAA Natural History Protocol and is referred to as AUD history in this study. Current alcohol consumption at the C19-PIA Study was assessed using the three-item Alcohol Use Disorders Identification Test Consumption (AUDIT-C; Bradley et al., 2007).

#### 2.2.3 Substance use behaviors

The Centers for Disease Control and Prevention (2020) created a COVID-19 Community Survey Question Bank which included survey items on substance use and coping behaviors during the pandemic. Items from this survey were adapted in the current study, where participants were asked: “As a result of the COVID-19 pandemic, are you doing any of the following?” Items involving substance use were included in the analyses, namely the following: (1) drinking alcohol, (2) smoking more cigarettes or vaping more, and (3) using cannabis or marijuana. Participants responded using a dichotomous “yes” or “no” endorsement.

#### 2.2.4 Positive coping behaviors

Positive coping behavior items were also adapted from the COVID-19 Community Survey Question Bank (Centers for Disease Control and Prevention, 2020). Four positive coping behaviors were included in the analyses, namely (1) taking care of your body, such as taking deep breaths, stretching, or meditating, (2) making time to relax, (3) connect with others, including talking with people you trust about your concerns and how you are feeling, and (4) engaging in healthy behaviors like trying to eat healthy, well-balanced meals, exercising regularly, getting plenty of sleep, or avoiding alcohol and drugs (McCabe et al., 2024). These items are respectively referred to as take care of body, make time to relax, connect with others, and multiple healthy behaviors hereafter. Participants responded using a dichotomous “yes” or “no” endorsement.

### 2.3 Statistical analysis

To address the first goal of this study, impact of AUD history on overall sleep quality from before to during the COVID-19 pandemic were evaluated using a multilevel generalized linear model, where two observations of PSQI scores were nested within individuals. To test whether changes in PSQI overall score varied by AUD history, the interaction between time and AUD was included in the model and probed if significant. To address the second goal of this study, associations of substance use and positive coping behaviors with seven domains of sleep quality were evaluated using linear regression models. All statistical analyses were conducted using Stata 18.

## 3 Results

The mean age of the study sample was 43.7 years (SD = 14.2). The study sample was 49.1% female, 56.8% White, 29.2% Black/African, and 13.9% Other Race. Most participants were Single (64.3%) and came from diverse backgrounds with a range of educational level and household income well represented (see Table 1). AUD history was positive in 37.3% of the study sample. Compared to participants who scored below 5 on the PSQI (31.4%), those who scored 5 or higher on the PSQI (68.6%) did not differ in any of the demographic characteristics evaluated. PSQI scores differed by AUD history and AUDIT-C scores. Among those with PSQI scores below 5, 24.8% had an AUD history, whereas among those with PSQI scores at or above 5, 43.0% had an AUD history (Odds Ratio = 2.29, 95% Confidence Intervals [CI]: 1.40, 3.72, *p* = 0.001). The overall sample had an average AUDIT-C score of 4.2 with a standard deviation of 3.9. Among those with PSQI scores below 5, the mean AUDIT-C score was 3.0 with a standard deviation of 3.4, whereas among those with PSQI scores at or above 5, the mean AUDIT-C score was higher at 4.7 with a standard deviation of 4.0 (*t* = 3.97, *p* < 0.001; Cohen's *d* = 0.44).

**Table 1:** Sample characteristics for the overall sample and by Pittsburgh Sleep Quality Index clinical threshold.

	**Overall (*****N*** = **373)**		**PSQI**<**5 (*****n*** = **117; 31.4%)**		**PSQI** ≥**5 (*****n*** = **256; 68.6%)**			
**Continuous variables**	**Mean**	**SD**	**Mean**	**SD**	**Mean**	**SD**	***t***	***p***
Age	43.7	14.2	43.4	14.3	43.9	14.2	0.32	0.747
AUDIT-C	4.2	3.9	3.0	3.4	4.7	4.0	3.97	< 0.001
**Categorial variables**	**Frequency**	**Percent**	**Frequency**	**Percent**	**Frequency**	**Percent**	*χ^2^*	***p***
Sex							0.01	0.928
Female	183	49.1%	57	48.7%	126	49.2%		
Male	190	50.9%	60	51.3%	130	50.8%		
Race							1.69	0.429
White	212	56.8%	61	52.1%	151	59.0%		
Black/African	109	29.2%	39	33.3%	70	27.3%		
Other^a^	52	13.9%	17	14.5%	35	13.7%		
Ethnicity							0.03	0.986
Not Hispanic	329	88.2%	103	88.0%	226	88.3%		
Hispanic	29	7.8%	9	7.7%	20	7.8%		
Unknown	15	4.0%	5	4.3%	10	3.9%		
Marital status							0.83	0.662
Single	240	64.3%	74	63.3%	166	64.8%		
Married	86	23.1%	30	25.6%	56	21.9%		
Other^b^	47	12.6%	13	11.1%	34	13.3%		
Years of education^c^							0.32	0.850
< 13	84	23.0%	25	21.7%	59	23.6%		
13–16	192	52.6%	60	52.2%	132	52.8%		
17 or higher	89	24.4%	30	26.1%	59	23.6%		
Annual household income^d^							0.91	0.635
< $20,000	85	23.2%	24	20.7%	61	24.3%		
$20,000–$74,999	157	42.8%	49	42.2%	108	43.0%		
$75,000 or more	125	34.1%	43	37.1%	82	32.7%		
Alcohol Use Disorder History							11.36	0.001
No	234	62.7%	88	75.1%	146	57.0%		
Yes	139	37.3%	29	24.8%	110	43.0%		
Study enrollment phase							4.49	0.343
I	72	19.3%	22	18.8%	50	19.5%		
II	103	27.6%	26	22.2%	77	30.1%		
III	91	24.4%	33	28.2%	58	22.7%		
IV	51	13.7%	20	17.1%	31	12.1%		
V	56	15.0%	16	13.7%	40	15.6%		
Years since Natural History Protocol							5.78	0.123
< 1 year	159	42.6%	47	10.2%	112	43.8%		
1 year	65	17.4%	19	16.2%	46	18.0%		
2–3 years	88	23.6%	24	20.5%	64	25.0%		
4 or more years	61	16.4%	27	23.1%	34	13.3%		

^*a*^Other racial groups included Asian (n = 27), multiracial (n = 7), American Indian/Alaska Native (n = 1), and unknown race (n = 17).

^*b*^Other marital status included divorced (n = 28), separated (n = 6), widowed (n = 5), other marital status (n = 1), and not provided (n = 7).

^*c*^Eight participants had missing data on years of education. Valid percentages are presented.

^*d*^Six participants had missing data on household income. Valid percentages are presented.

**Figure 1:**
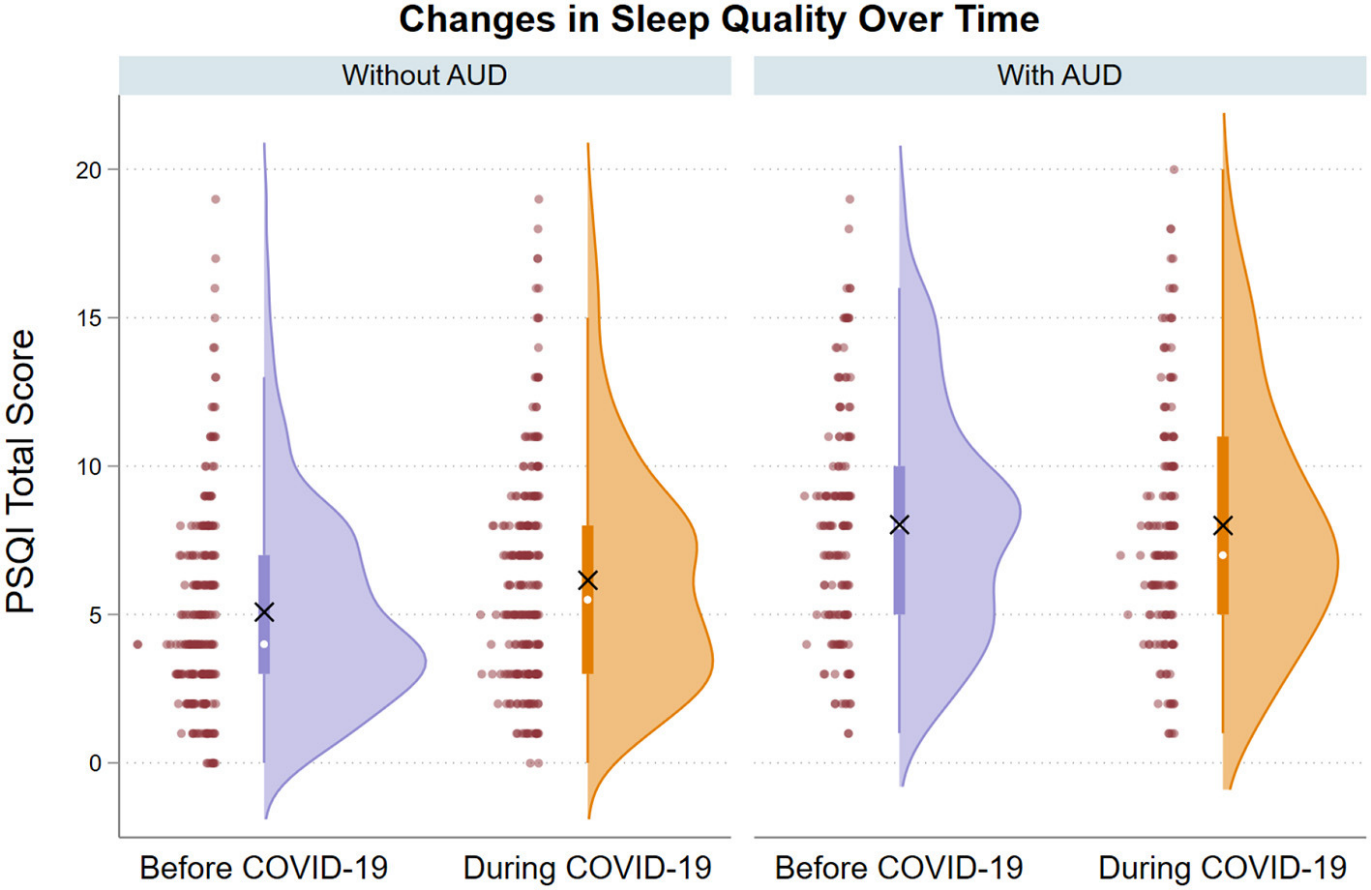
Raincloud plots showing PSQI total scores from before to during COVID-19 by history of AUD. In the raincloud plots, the distribution of the data is shown with data points to the left and a half violin plot to the right. The white dot represents the median and the X symbol represents the mean.

Total PSQI scores before the COVID-19 pandemic differed based on AUD history (Figure 1). On average, PSQI score before COVID-19 among those without AUD history was 5.09 (95% CI: 4.66, 5.52), whereas PSQI score among those with AUD history was 8.04 (95% CI: 7.38, 8.69). During the pandemic, average PSQI score among those without AUD history was 6.16 (95% CI: 5.68, 6.64) and average PSQI score among those with AUD history was 8.01 (95% CI: 7.32, 8.70). Individuals without AUD history exhibited an increase in PSQI scores (*b* = 1.07, 95% CI: 0.62, 1.53) from before to during the pandemic, whereas those with AUD history exhibited no statistically significant change (*b* = −0.03, 95% CI: −0.62, 0.56), which may be due to the higher PSQI scores in the AUD group that pre-existed before COVID-19.

**Figure 2:**
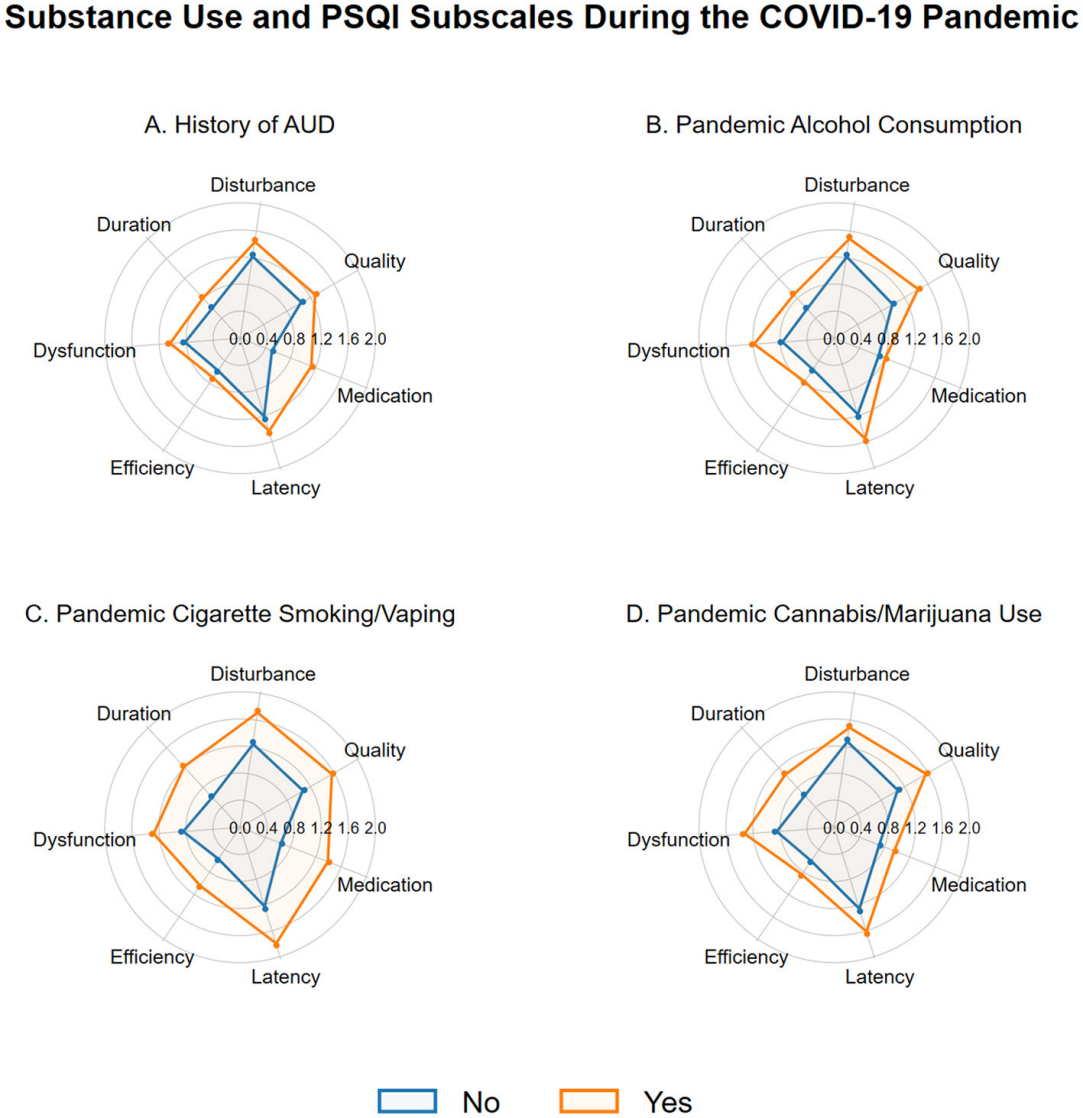
Radar charts showing associations between pandemic substance use and PSQI subscale scores. **(A)** History of AUD. **(B)** Pandemic alcohol consumption. **(C)** Pandemic cigarette smoking/vaping. **(D)** Pandemic cannabis/marijuana use.

Associations of substance use and positive coping behaviors with seven PSQI subscales during the pandemic are presented in Table 2, and the corresponding effect sizes are presented in Table 3. AUD history was associated with worse sleep disturbance (*b* = 0.22), sleep quality (*b* = 0.23), sleep latency (*b* = 0.22), daytime dysfunction (*b* = 0.22), and higher use of medication to sleep (*b* = 0.63), with the largest impact on medication (Figure 2A). The overall proportion of the study sample who endorsed using any sleep medication was 39.4%, and endorsement of any sleep medication use during the past month was higher among participants with AUD than those without (51.1% vs. 32.5%, χ^2^ = 12.6, *p* < 0.001). Endorsement of alcohol consumption as a result of the pandemic (31.4% of the study sample) was associated with worse sleep duration (*b* = 0.29), sleep disturbance (*b* = 0.27), sleep quality (*b* = 0.44), sleep latency (*b* = 0.37), sleep efficiency (*b* = 0.22), and daytime dysfunction (*b* = 0.42; Figure 2B). Endorsement of cigarette smoking/vaping (12.3% of the study sample) was associated with worsening of all PSQI subscales (*b* ranged from 0.43 to 0.75; Figure 2C). Endorsement of cannabis/marijuana use (9.9% of the study sample) was associated with worsened sleep duration (*b* = 0.42), sleep quality (*b* = 0.48), sleep latency (*b* = 0.35), and daytime dysfunction (*b* = 0.47; Figure 2D), possibly reflecting bi-directional associations between cannabis/marijuana use and sleep issues.

**Table 2:** Associations of substance use and positive coping behaviors with PSQI sleep quality subscales during the pandemic.

	**Duration**	**Disturbance**	**Quality**	**Medication**	**Latency**	**Efficiency**	**Dysfunction**
	***b*** **(95% CI)**	***b*** **(95% CI)**	***b*** **(95% CI)**	***b*** **(95% CI)**	***b*** **(95% CI)**	***b*** **(95% CI)**	***b*** **(95% CI)**
**Substance use behaviors**							
History of AUD	0.20 (−0.00, 0.41)	**0.22 (0.09, 0.35)**	**0.23 (0.03, 0.42)**	**0.63 (0.40, 0.85)**	**0.22 (0.00, 0.43)**	0.13 (−0.07, 0.32)	**0.22 (0.05, 0.40)**
Alcohol consumption	**0.29 (0.08, 0.50)**	**0.27 (0.13, 0.40)**	**0.44 (0.24, 0.64)**	0.10 (−0.14, 0.34)	**0.37 (0.15, 0.60)**	**0.22 (0.01, 0.42)**	**0.42 (0.24, 0.60)**
Smoking/vaping	**0.62 (0.32, 0.91)**	**0.47 (0.28, 0.65)**	**0.49 (0.20, 0.77)**	**0.75 (0.42, 1.08)**	**0.56 (0.25, 0.88)**	**0.48 (0.19, 0.76)**	**0.43 (0.18, 0.69)**
Cannabis/marijuana use	**0.42 (0.09, 0.75)**	0.20 (−0.00, 0.41)	**0.48 (0.17, 0.80)**	0.24 (−0.13, 0.61)	**0.35 (0.00, 0.70)**	0.24 (−0.07, 0.56)	**0.47 (0.19, 0.75)**
**Positive coping behaviors**							
Take care of body	−0.16 (−0.37, 0.05)	**−0.14 (−0.27**, **−0.01)**	**−0.36 (−0.56**, **−0.16)**	−0.20 (−0.43, 0.04)	−0.11 (−0.34, 0.11)	−0.15 (−0.36, 0.05)	−0.11 (−0.29, 0.07)
Make time to relax	**−0.51 (−0.79**, **−0.22)**	**−0.30 (−0.48**, **−0.12)**	**−0.38 (−0.66**, **−0.11)**	**−0.44 (−0.76**, **−0.12)**	−0.11 (−0.42, 0.19)	−0.25 (−0.53, 0.02)	−0.11 (−0.36, 0.14)
Connect with others	−0.13 (−0.38, 0.12)	−0.14 (−0.30, 0.02)	−0.08 (−0.32, 0.16)	**−0.42 (−0.70**, **−0.14)**	−0.02 (−0.28, 0.25)	**−0.25 (−0.49**, **−0.01)**	0.03 (−0.19, 0.24)
Multiple healthy behaviors	**−0.59 (−0.84**, **−0.33)**	**−0.40 (−0.56**, **−0.24)**	**−0.61 (−0.85**, **−0.37)**	**−0.39 (−0.68**, **−0.09)**	**−0.40 (−0.67**, **−0.13)**	**−0.41 (−0.66**, **−0.16)**	**−0.39 (−0.61**, **−0.17)**

Linear regression coefficients along with the 95% confidence intervals are presented. Significant associations (*p* < 0.05) are shown in bold.

**Table 3:** Effect sizes of the associations of substance use and positive coping behaviors with PSQI sleep quality subscales.

	**Duration**	**Disturbance**	**Quality**	**Medication**	**Latency**	**Efficiency**	**Dysfunction**
	**Cohen's** ***d***	**Cohen's** ***d***	**Cohen's** ***d***	**Cohen's** ***d***	**Cohen's** ***d***	**Cohen's** ***d***	**Cohen's** ***d***
**Substance use behaviors**							
History of AUD	0.21	0.36	0.25	0.60	0.21	0.14	0.27
Alcohol consumption	0.30	0.44	0.49	0.09	0.37	0.23	0.52
Smoking/vaping	0.64	0.78	0.53	0.70	0.56	0.52	0.53
Cannabis/marijuana use	0.44	0.33	0.53	0.22	0.34	0.26	0.58
**Positive coping behaviors**							
Take care of body	0.16	0.23	0.39	0.18	0.11	0.16	0.13
Make time to relax	0.53	0.49	0.42	0.41	0.11	0.27	0.13
Connect with others	0.13	0.23	0.09	0.39	0.02	0.27	0.03
Multiple healthy behaviors	0.62	0.67	0.67	0.36	0.39	0.45	0.47

Effect sizes can be interpreted as small (*d* = 0.20), medium (*d* = 0.50), and large (*d* = 0.80).

**Figure 3:**
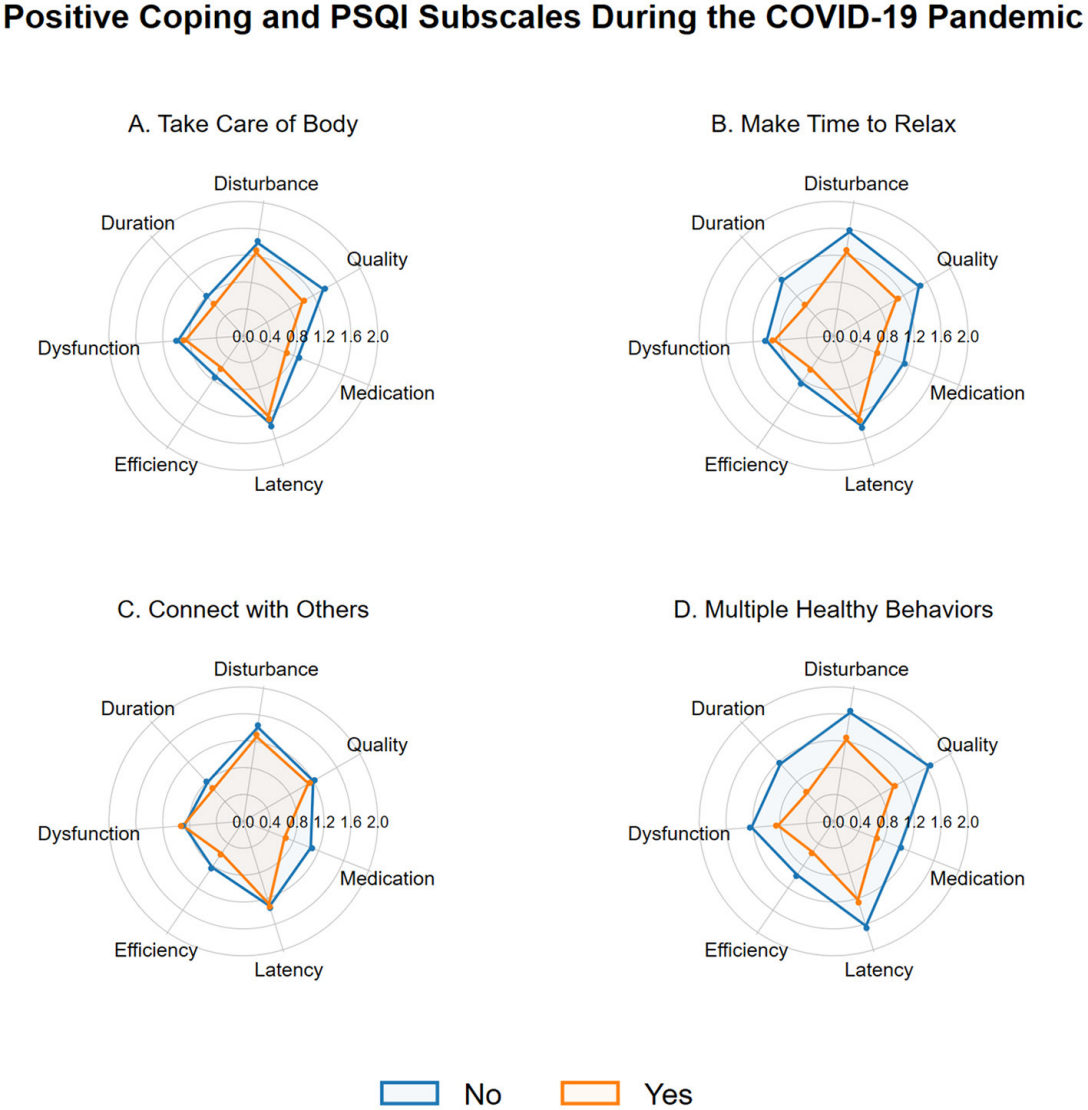
Radar charts showing associations between pandemic positive coping and PSQI subscale scores. **(A)** Take care of body. **(B)** Make time to relax. **(C)** Connect with others. **(D)** Multiple healthy behaviors.

In terms of positive coping behaviors during the pandemic, 67.8% endorsed taking care of their body, 86.3% endorsed making time to relax more, 80.7% endorsed connecting with others more, and 82.6% endorsed engaging in multiple healthy behaviors. Associations between positive coping behaviors and seven PSQI subscales during the pandemic are presented in Table 2. Endorsement of take care of body was associated with less sleep disturbance (*b* = −0.14) and better sleep quality (*b* = −0.36; Figure 3A). Endorsement of make time to relax was associated with better sleep duration (*b* = −0.51), less sleep disturbance (*b* = −0.30), better sleep quality (*b* = −0.38), and decreased use of medication to sleep (*b* = −0.44; Figure 3B). Endorsement of connect with others was associated with decreased use of medication to sleep (*b* = −0.42) and better sleep efficiency (*b* = −0.25; Figure 3C). Endorsing multiple healthy behaviors showed consistent associations with better scores for all sleep domain subscales (*b* ranged from −0.61 to −0.39; Figure 3D).

## 4 Discussion

Studies have shown that sleep problems associated with AUD manifest during the stages of intoxication, withdrawal, and into acute and prolonged abstinence (Koob and Colrain, 2020). Consistent with these findings, we found that AUD history was associated with higher PSQI scores, or worse sleep quality, both before and during the pandemic. Contrary to our hypothesis, an increase in PSQI score was observed not among individuals with AUD history, but only among those without AUD history. As shown in the raincloud plots, individuals with AUD history may have worse sleep quality to begin with and the disparity by AUD status persisted during the COVID-19 pandemic. These findings are similar to the pattern of results observed for quality-of-life measures, where worse quality of life prior to the pandemic may have limited further decline during the pandemic (Luk et al., 2023a). On the other hand, the significant increase in PSQI scores among those without AUD history may reflect general population trends toward worsened sleep quality during the pandemic (Jahrami et al., 2021).

When examining the associations between AUD history and PSQI sleep domains, the largest difference was found in the use of medication for sleep. Prior literature has revealed that a commonly endorsed function of alcohol is to bring about sleep relief (Lac and Luk, 2023; Miller et al., 2021). Considered within the context of a stressful public health crisis, this finding may support the idea that those with substance use problems are more likely to use drug solutions to cope (Khantzian, 1985). Despite the prevalence of sleep disturbance among those in recovery from AUD, physicians may be reluctant to prescribe medication to treat sleep disturbance in recovering patients (Friedmann et al., 2003). More clinical research is needed to further our understanding of when and for whom sleep medications may be appropriate for individuals recovering from AUD and the complementary role of behavioral sleep interventions. In addition, AUD history was associated with worse sleep disturbance, sleep quality, sleep latency, and daytime dysfunction. These findings are consistent with prior literature demonstrating that alcohol misuse may negatively impact sleep quality (Koob and Colrain, 2020; Chakravorty et al., 2016; Colrain et al., 2014).

We also found significant associations between different types of substance use and sleep quality during the pandemic. Alcohol consumption due to the pandemic was associated with worse scores on six out of the seven PSQI sleep domains, namely sleep duration, sleep disturbance, sleep quality, sleep latency, sleep efficiency, and daytime dysfunction. Alcohol consumption has significant negative implications for psychological wellbeing. An online survey administered to US adults in 2020 found that increased drinking habits, including binge drinking, during the pandemic was associated with higher likelihood of mental health disorders (Yue et al., 2023). The associations between sleep quality, mental health, and alcohol consumption are complex and represent a crucial component of health to consider within the context of a stress-inducing situation such as the COVID-19 pandemic.

Cigarette smoking/vaping was a type of substance use behavior that was most consistently associated with all seven sleep domains. Smoking cigarettes has been associated with an increase in sleep problems (Purani et al., 2019; Cohrs et al., 2014). A study using polysomnography to evaluate differences in sleep quality between smokers and non-smokers found insomnia-like disruptions amongst smokers, including shorter sleep duration, and increased sleep latency (Jaehne et al., 2012). Nicotine dependence, including dependence upon electronic cigarettes, has also been associated with poorer sleep quality (Zvolensky et al., 2020; Dugas et al., 2017). Our study extends these findings by demonstrating the impact of cigarette smoking/vaping on all sleep domains of the PSQI. These findings highlight the need to target smoking cessation in interventions for improving sleep quality.

Cannabis use is often driven by coping motives, which also has negative implications for psychological wellbeing (Glodosky and Cuttler, 2020; Brodbeck et al., 2007). Our study revealed that cannabis/marijuana use due to the pandemic was associated with worse scores on sleep duration, sleep quality, sleep latency, and daytime dysfunction. Our findings are consistent with recent data showing that cannabis use increased at the early phase of the pandemic (Brenneke et al., 2022) and may reflect an increase in boredom motives during the pandemic (Graupensperger et al., 2021). In an epidemiologic study of US adults, cannabis use increased by 91% among those with mental health conditions reporting medicinal cannabis use (Vidot et al., 2021). In a recent study, perceived beliefs about the use of cannabis as a sleep aid was associated with increased cannabis use (Graupensperger et al., 2023). Taken together, these studies highlight the potential of addressing beliefs or motives related to cannabis use in the context of cannabis use prevention and intervention.

Positive coping behaviors—take care of body, make time to relax, connect with others, and multiple healthy behaviors—were associated with better sleep in selected domains. These findings underscore the possibility of utilizing strength- and resilience-based approaches to improve sleep quality during a stressful time. Interestingly, Multiple Healthy Behaviors was the only positive coping behavior associated with lower PSQI scores across all seven domains. Of note, this measure of positive coping listed several healthy behaviors together and included “getting plenty of sleep” as a healthy behavior which may conflate the observed associations and limit the interpretation due to the conceptual overlap. That said, these associations may also point to the need to alter multiple lifestyle behaviors to support improvement in sleep quality. Future research can disaggregate the various components assessed by this item and enhance our understanding of the connections between multiple healthy behaviors and sleep quality domains.

A strength of this study was that it included a sample of individuals with COVID-19 data as well as relevant data from the NIAAA Natural History Protocol. As such, we were able to examine changes in sleep quality as a result of the pandemic amongst those with and without AUD history. Furthermore, this study examined specific sleep domains and multiple types of substance use. However, this study is not without limitations. First, assessment of sleep quality and substance use was based on self-report, and the PSQI was administered twice within the same survey to evaluate pre-pandemic and pandemic sleep quality levels. Retrospective report of pre-pandemic sleep quality may be vulnerable to self-report bias and the repeated administrations of the PSQI within the same assessment may be a potential confounding factor. Furthermore, the PSQI was validated to assess sleep quality in the past month and so adapting it to assess pre-pandemic sleep quality that could be several months ago may introduce recall bias. To address these methodological issues, future research can include objective measures of sleep and utilize longitudinal data collected across multiple timepoints. Second, positive coping and substance use behaviors were assessed using a binary response format and did not capture the frequency of these behaviors. Third, analyses related to the second goal were cross-sectional in nature and so the direction of effects cannot be ascertained. Fourth, given the primary goal of the larger study focused on drinking and substance use behaviors during the pandemic, data on history of sleep disorders and sleep medication use were not available. Fifth, the sample size was limited and might not fully capture the diversity of COVID-related stressors. Despite these limitations, this study has meaningful clinical implications as sleep health is a marker for general wellbeing and can be addressed by promoting sleep hygiene practices and/or using evidence-based treatments such as cognitive behavioral therapy or mindfulness-based interventions (Irish et al., 2015; Chan et al., 2021; Hertenstein et al., 2022; Luk and Thompson, 2024; Rusch et al., 2019; Peters et al., 2022; Gross et al., 2011; Fu et al., 2022; Black et al., 2015).

In conclusion, we found that AUD history, substance use, and positive coping behaviors were significantly associated with sleep quality during the pandemic. AUD history, alcohol consumption, cannabis/marijuana use, and cigarette smoking/vaping were associated with worse sleep quality, whereas taking care of body, making time to relax, connecting with others, and multiple healthy behaviors were associated with better sleep quality. These insights can inform future therapeutic targets that can be addressed using evidence-based treatments (e.g., cognitive behavioral therapy and mindfulness practices) within the context of significant stress exposure. Future research can examine how implementation of sleep hygiene practices and other behavioral therapies may help alleviate the burden associated with poor sleep quality, support substance use recovery, and improve individuals' overall health and wellbeing.

## Data Availability

The datasets presented in this article are not readily available due to ethical concerns regarding patient privacy and original patient consent. Data may be made available by requests directly to the corresponding authors. Requests to access the datasets should be directed to jeremy.luk@nih.gov or vijayr@mail.nih.gov.
